# Contributions of *Fusarium virguliforme* and *Heterodera glycines* to the Disease Complex of Sudden Death Syndrome of Soybean

**DOI:** 10.1371/journal.pone.0099529

**Published:** 2014-06-16

**Authors:** Andreas Westphal, Chunge Li, Lijuan Xing, Alan McKay, Dean Malvick

**Affiliations:** 1 Julius Kühn-Institut, Institute for Plant Protection in Field Crops and Grassland, Braunschweig, Germany; 2 Department of Botany and Plant Pathology, Purdue University, West Lafayette, Indiana, United States of America; 3 State Key Laboratory of Environmental Aquatic Chemistry, Research Center for Eco-Environmental Sciences, Chinese Academy of Sciences, Beijing, China; 4 Marrone Bio Innovations Inc., Davis, California, United States of America; 5 South Australian Research and Development Institute, Plant Research Centre, Urrbrae, South Australia, Australia; 6 Department of Plant Pathology, University of Minnesota, St. Paul, Minnesota, United States of America; Agriculture and Agri-Food Canada, Canada

## Abstract

**Background:**

Sudden death syndrome (SDS) of soybean caused by *Fusarium virguliforme* spreads and reduces soybean yields through the North Central region of the U.S. The fungal pathogen and *Heterodera glycines* are difficult to manage.

**Methodology/Principal Findings:**

The objective was to determine the contributions of *H. glycines* and *F. virguliforme* to SDS severity and effects on soybean yield. To quantify DNA of *F. virguliforme* in soybean roots and soil, a specific real time qPCR assay was developed. The assay was used on materials from soybean field microplots that contained in a four-factor factorial-design: (i) untreated or methyl bromide-fumigated; (ii) non-infested or infested with *F. virguliforme*; (iii) non-infested or infested with *H. glycines*; (iv) natural precipitation or additional weekly watering. In years 2 and 3 of the trial, soil and watering treatments were maintained. Roots of soybean ‘Williams 82’ were collected for necrosis ratings at the full seed growth stage R6. Foliar symptoms of SDS (area under the disease progress curve, AUDPC), root necrosis, and seed yield parameters were related to population densities of *H. glycines* and the relative DNA concentrations of *F. virguliforme* in the roots and soil. The specific and sensitive real time qPCR was used. Data from microplots were introduced into models of AUDPC, root necrosis, and seed yield parameters with the frequency of *H. glycines* and *F. virguliforme*, and among each other. The models confirmed the close interrelationship of *H. glycines* with the development of SDS, and allowed for predictions of disease risk based on populations of these two pathogens in soil.

**Conclusions/Significance:**

The results modeled the synergistic interaction between *H. glycines* and *F. virguliforme* quantitatively in previously infested field plots and explained previous findings of their interaction. Under these conditions, *F. virguliforme* was mildly aggressive and depended on infection of *H. glycines* to cause highly severe SDS.

## Introduction

Many diseases negatively impact soybean (*Glycine max* (L.) Merr.) production in major cropping areas [1]. In the top eight soybean-producing countries, sudden death syndrome (SDS) of soybean occurs frequently [1]. In North America, this disease is caused by *Fusarium virguliforme* Aoki et al. [2], formerly *F. solani* (Mart.) Sacc. f. sp. *glycines* [3], [4]. In South America, the disease can be caused by several distinct species of *Fusarium* including *F. brasiliense, F. cuneirostrum, F. tucumaniae, F*. *virguliforme*, and more recently, *F. crassistipitatum* [5], [6].The disease has increased in distribution and economic importance in the Midwest of the US [1], [7], [8], [9]. *Fusarium virguliforme* colonizes the roots and produces phytotoxins that are translocated to the tops of plants where they cause foliar chlorosis and necrosis [8], [10], and in severe cases, premature defoliation can result [8]. The fungal pathogen interacts with other soil-borne plant pathogens, of which *Heterodera glycines* Ichinohe (soybean cyst nematode; SCN) is the most common and widely recognized synergist [11], [12], [13]. While *Heterodera glycines* alone causes significant yield losses in most areas of soybean production in the U.S., [1], [7], [14], the synergistic interaction on a susceptible cultivar with *F. virguliforme* [15] appears to result in greater damage and requires additional efforts from plant pathologists to develop plant disease suppressing strategies. Management tactics for SDS and SCN emphasize choosing resistant cultivars and implementing cultural practices [16]. Several breeding lines with elevated resistance against SDS have been identified and released, e.g., ‘Saluki 4411’ [17]. Cultivars with resistance to at least one of the pathogens can have yield advantages and mitigating effects on SDS [13]. The management of SCN with crop rotation can be effective, but the crop rotation widespread in the North Central Region of the US of soybean with corn has not reduced SDS [18]. Some opportunities have been seen in the development of suppressive soils of this disease complex [19], but additional studies of this have not been pursued.

The interactions of plant-parasitic nematodes with various soil-borne fungal pathogens to result in high levels of disease on the plant hosts have been demonstrated [11], [20], [21], [22]. Synergism in causing severe SDS by co-infection with *H. glycines* and *F. virguliforme* was demonstrated [15] but the mechanism and quantitative contributions within the interaction of *H*. *glycines* and *F. virguliforme* have not been fully elucidated. Contrary to the synergistic effects with SCN on the plant, an antagonistic role of *F. solani* isolates against eggs of *H. glycines* [23] was reported by McLean and Lawrence [24]. However, this is currently discounted because it is likely that *F. virguliforme* is distinct from the *F. solani* that was pathogenic to the nematode eggs [25].

Studying the interaction between *F. virguliforme* and *H. glycines* is hampered by methodology limitations and challenges with reproducing field results in controlled greenhouse studies. For example, Gao and coworkers did not observe an interaction between the two pathogens under greenhouse conditions [26]. In-depth studies of the interaction in microplot experimentation in the field have generated reproducible results, and are key experimental contexts to understand the interactive contributions of the two pathogens to the disease complex [11], [12], [15]. Accurate measurement methods for the determination of severity classes of fungal infection are necessary to improve the evaluation of management inputs and resistance testing because of the high environmental impact on disease development. Fungal population density determinations from soil and plants have primarily relied on the use of a semi-selective modified Nash and Snyder's medium [27], [28]. However, dilution plating can be tedious, and generating accurate and precise numbers is challenged by the shortcomings in selectivity of the medium and slow growth habit of *F. virguliforme*. Because of similar problems in other pathogen systems, PCR methods have been developed for improved detection and quantification, and they have been used to generate information on infection levels for plant disease development and predictions [29]. An example of coupling quantitative real-time PCR (qPCR) assays with extraction of DNA from soil is given with a DNA-based testing system offered in Australia to quantify soil-borne pathogens and predict risk for plant disease [30].

Several sets of PCR primers aiming at amplification of a mitochondrial gene or a tox gene of *F. virguliforme* have been developed [31], [32], [33]. These primers and the accompanying qPCR assays have primarily been used for specific amplification in plants grown under controlled conditions, but those available at the time this research was done were not specific for *F. virguliforme* [5]. A more robust and specific method for detection and quantification of DNA from *F. virguliforme* for field research was urgently needed.

The primary objective of this study was to determine the interactive contributions of *H. glycines* and *F. virguliforme* on SDS development and severity and soybean seed yield. The secondary goal was to develop a qPCR assay for specific detection and quantification of *F. virguliforme* in field-grown soybean roots and soil for this and other studies. The interactive contributions of *F. virguliforme* and *H. glycines* on foliar SDS development, root disease severity, and on soybean seed yield were modeled.

## Materials and Methods

### Development of a specific qPCR assay for F. virguliforme

In search of a suitably unique target DNA region within *Fusarium virguliforme* (Fv), the intergenic spacer (IGS) region was searched for possible primer sequences. Forward and reverse primers (FvIGS-F1 and FvIGS-R3, respectively) and a TaqMan probe (named FvIGS-Probe2; with reporter  =  FAM and quencher  =  TAMRA) were developed for qPCR analysis of *F. virguliforme*. The sequence of the forward primer FvIGS-F1 was: 5′-GGTGGTGCGGAAGGTCT-3′ and the sequence of the reverse primer FvIGS-R3 was: 5′-CCCTACACCTTTCGTACCAT-3′. The sequence for the FvIGS-Probe2 probe was selected by manual annotation using Primer Express v.3.0 software (Applied Biosystems [ABI], Foster City, CA). The probe sequence was: 6FAMATAGGGTAGGCGGATCTGACTTGGCGTAMRA. The TaqMan probe was obtained from ABI.

Amplification and detection of Fv DNA with real time, quantitative (qPCR) was conducted with an ABI 7500 sequence detection system. Initial testing of the qPCR assay was run with SYBR Green detection. The reactions were performed in a volume of 25 µL which included 2 µL of 10-fold diluted genomic DNA, 12.5 µL 2×SYBR Green Master Mix (ABI), 600 nM each of the forward and reverse primers, and 7.5 µL purified water. The reaction mixtures were amplified at 50°C for 2 min and 95°C for 10 min, followed by 40 cycles of 95°C for 15 s and 67°C for 1 min. Following the initial testing, qPCR assays in this study were conducted with a TaqMan probe-based assay as follows. Genomic DNA samples were diluted 1∶10 with pure H_2_0 for qPCR analysis. Reactions were conducted in a volume of 25 µl that contained 5 µl of the DNA sample, 12.5 µl TaqMan Gene Expression master mix (ABI), 200 nM probe, 450 nM each of the forward and reverse primers, and 2.75 µl of molecular grade water. Electronic pipettes (Rainin Instrument, LLC, Woburn, MA) were used to minimize variability in volume delivery to reaction wells. Thermal cycling parameters consisted of 2 min at 50°C and 10 min at 95°C, followed by 40 cycles of 15 s at 95°C and 1 min at 66°C. Reactions were run in duplicate on individual plates, and analyzed based on absolute quantification using ABI software. To check for cross contamination, DNase/RNase-treated water samples were included in all experiments as non-template controls. Equipment was decontaminated with the product “DNA Away” (Molecular BioProducts, San Diego, CA) and aerosol barrier tips were used for all pipetting to minimize cross contamination. Samples of Fv DNA of known concentrations were included on all reaction plates as positive controls and for comparison of quantification among plates.

### Quantification of DNA of F. virguliforme from pure cultures and soil

Using a Quant-iT dsDNA Broad Range Assay Kit and Qubit fluorometer (Molecular Probes, Eugene, OR), the DNA concentration of a *F. virguliforme* sample from pure culture of isolate BE3ss6 was determined [33]. Serial dilutions of this sample, where the highest concentration DNA was 2.85 µg ml^−1^ were analyzed using qPCR as described above to develop a standard curve. In a second step, a standard curve for detection of Fv DNA in soil was developed as follows. Soil with negligible presence of Fv used in the microplots was sieved, 500 g samples were spiked with a ten-fold concentration series of *F. virguliforme* at 1×10^4^ to 1×10^7^ macroconidia per 100 g of soil. The spiked soil samples were sent for DNA extraction by the Root Disease Testing Service (SARDI, SA Australia) [29], and DNA shipped back to the U.S. for evaluation with the qPCR assay. The entire process was conducted twice with every soil sample and spore infestation level, and the qPCR assay was conducted in two technical replications for each sample.

### Specificity of qPCR tested against other soil-borne fungi and fungal-like organisms

Specificity of the qPCR assay was tested with DNA extracted from closely related *Fusarium* spp. and a range of other fungi that could occur in soybean roots and soybean field soil (Table 1, 2). The identity of the *Fusarium* spp. isolates was confirmed with analysis of morphological features and by analysis of translation elongation factor 1-alpha DNA sequences [34], [35]. The other genera and species were identified by analysis of morphological features and ITS DNA sequences. In addition, an *in silico* assay was conducted to determine the theoretical potential for annealing of the primer sequences with established DNA sequences from other soybean pathogens. Queries were conducted for each of the F1 and the R3 sequences on the NCBI database website (http://blast.ncbi.nlm.nih.gov/Blast.cgi?PROGRAM=blastn&BLAST_PROGRAMS=megaBlast&PAGE_TYPE=BlastSearch&SHOW_DEFAULTS=on&LINK_LOC=blasthome). The primary search focused on IGS sequences, and a secondary search included other regions of the fungal genomes.

**Table 1:** *Fusarium* species used to test specificity of an IGS sequence-based SYBR green or TaqMan qPCR assay designed to detect *Fusarium virguliforme*.

Genus and species	Isolate^a^	Ct value
*Fusarium acuminatum*	07-455^a^	36.6
*F. acuminatum*	07-461^a^	34.7
*F. brasiliense*	NRRL 31757^b^	35.9
*F. brasiliense*	P-22734	>39
*F. cuneirostrum*	NRRL 31157^b^	>39
*F. cuneirostrum*	E-31949	>39
*F. equiseti*	07-456^a^	36.2
*F. equiseti*	07-465^a^	35.6
*F. graminearum*	07-351^a^	34.7
*F. graminearum*	07-450^a^	36.1
*F. oxysporum*	AW101^b^	>39
*F. oxysporum*	AW104^b^	>39
*F. oxysporum*	07-390^a^	36.9
*F. oxysporum*	07-393^a^	35.4
*F. oxysporum*	07-317^a^	33.9
*F. oxysporum*	07-335^a^	36.5
*F. phaseoli*	NRRL 31156^b^	35.4
*F. proliferatum*	07-168^a^	36.3
*F. proliferatum*	07-175^a^	37.2
*F. proliferatum*	07-262^a^	34.9
*F. redolens*	07-313^a^	35.6
*F. redolens*	07-350^a^	36.3
*F. solani*	AW102^b^	>39
*F. solani*	07-379^a^	34.9
*F. solani*	07-388^a^	35.7
*F. solani*	MN 5022 62-41^a^	>39
*F. solani*	MN 5018 62-25^a^	38.5
*F. solani*	MN 000149-37^a^	>39
*F. solani*	MN 5023 62-45^a^	37.1
*F. solani*	91-110-3^a^	>39
*F. solani*	91-284-4^a^	>39
*F. solani*	91-79-1^a^	>39
*F. solani*	91-121-2^a^	>39
*F. solani*	91-92-1^a^	>39
*F. solani*	08-32	>39
*F. solani*	08-49	>39
*F. solani*	NF78	>39
*F. solani*	07-373	>39
*F. solani*	62	>39
*F. solani*	64	>39
*F.solani*	AW103^b^	>39
*F. sporotrichioides*	07-356^a^	37.2
*F. sporotrichioides*	07-361^a^	36.8
*F. subglutinans*	08-7^a^	34.3
*F. subglutinans*	08-34^a^	35.2
*F. tucumaniae*	NRRL 31096^b^	>39
*F. tucumaniae*	A-31773	39.3
*F. tucumaniae*	H-31096	35.4
*F. virguliforme*	AW107^b^	16.4
*F. virguliforme*	AW108^b^	19.2
*F. virguliforme*	NRRL 31041^b^	25.7
*F. virguliforme*	MN-B (check)	13.8
*F. oxysporum*	08-16	>39
*F. oxysporum*	08-19	>39
*F. oxysporum*	08-36	>39
*F. oxysporum*	07-071	>39
*F. oxysporum*	75	>39
*F. oxysporum*	08-35	>39
*Fusarium sp.*	NRRL 31949^b^	>39

Total DNA was extracted from pure cultures of each of these organisms and detection was assessed as cycle threshold (Ct) values generated with qPCR analysis.

a Samples of all isolates were 1∶10 dilutions of a 1x DNA extract sample unless otherwise noted.

b Samples were processed as described previously but SYBR green Master Mix was used for qPCR detection.

**Table 2:** Fungal genera and species expected in soybean tissue and soybean production soils used to test specificity of an IGS sequence-based TaqMan qPCR assay designed to detect *Fusarium virguliforme*.

Genus and species	Isolate^a^	Ct value
*Bionectria ochroleuca*	MN-1	>39
*B. ochroleuca*	NF5	>39
*Diaporthe phaseolorum* var. *caulivora*	Redwood10	>39
*Macrophomina phaseolina*	CR04B	>39
*Phialophora gregata, genotype A*	98G1-3	>39
*P. gregata,genotype B*	S2-1	>39
*Phomopsis longicolla*	Phom IA17	36.73
*Phytophthora sojae*	PsWI-16	>39
*Pythium dissotocum*	MN24JJ	>39
*P. longandrum*	RM	>39
*Rhizoctonia solani*	McLeod	>39
*R. solani*	5/7 Windels	>39
*R. solani*	Waseca	>39
*R. solani*	1B	>39
*R. solani*	NF50	>39
*Sclerotinia sclerotiorum*	Rosemount	>39
*Trichoderma sp.*	MN	>39

Total DNA was extracted from pure cultures of each of these organisms and detection was assessed as cycle threshold (Ct) values generated with qPCR analysis.

a Samples of all isolates were 1∶10 dilutions of a 1x sample unless otherwise noted.

### Microplot experiment

Microplots were established by inserting polyethylene tubes (45-cm-diameter) vertically 55-cm deep into a field at the Purdue University Agronomy Center for Research and Education, West Lafayette, IN (N 40.4706757, W 86.9926694) following standard procedures at this University research farm. Plots were filled with a sandy loam soil (65% sand; 21% silt; 14% clay; pH 6.7; 2.1% O.M.) from a naturally *H. glycines*–infested field with unknown status of SDS. In 2005, the following treatments were arranged with a 4-factor factorial design in a randomized complete block design with four replications: (i) non-treated or methyl bromide-fumigated at 450 kg ha^−1^; (ii) amended with 400 g of sterile sand- cornmeal (SC) mixture or with 400 g of *F. virguliforme* SC inocula; (iii) amended with 500 g of greenhouse substrate free of *H. glycines* or with 500 g of greenhouse substrate containing *H. glycines*; (iv) natural precipitation or additional 15 L of water added once a week starting at the R1 growth stage (beginning flowering; [36]). Before planting and at harvest, soil samples from plots were analyzed for SCN populations via cyst extractions using elutriation [37], and after counting, the cysts were crushed with a modified cyst crusher [38] to enumerate SCN eggs. Additional details for materials and methods for establishing the microplot experiment have been reported [15].

In 2006 and 2007, plots were maintained in the same treatments as assigned in 2005, and planted to soybean ‘Williams 82’ (susceptible to *F. virguliforme* and *H. glycines*) in three parallel rows consisting of two side rows of five plants and a central row of ten plants as done in 2005 [15]. From the onset of disease until the start of defoliation, severity of foliar SDS symptoms was determined every eight days on a scale of 0 (no disease) to 9 (plant succumbed to SDS) as described previously [39]. At growth stage R6, plants from the two side rows of each plot were dug out and washed for assessment of root necrosis based on a scale from 1 (no necrosis symptoms) to 5 (entirely necrotic; [12]). After rating, root systems were processed for DNA extraction as follows. Roots were washed thoroughly, fibrous and lateral roots were removed and a 5-cm section of each tap root originating from just below the soil line was excised. These root sections were scraped clean of soil and dead tissue, and surface-disinfested in a 0.6%-NaOCl solution for 1 minute. After drying on paper towels for 30 seconds, root pieces were soaked in autoclaved demineralized water for four minutes. The root pieces were then dried on clean paper towels for at least 30 seconds before the subsamples from each plot were combined and bagged in two layers of cheese cloth and lyophilized or kept at −80°C until lyophilizing.

### DNA extraction from root tissue and soil and quantification of F. virguliforme DNA

Lyophilized root samples were stored at −80°C and then ground in a Wiley Mill. Subsamples (100 mg) of the dried and ground plant root samples were used for DNA extraction following the extraction protocol by Malvick and Grunden [40]. Soil samples from the microplots were air-dried, portioned to 500 g subsamples, and sent to SARDI in Adelaide, Australia for extraction of total soil DNA by a proprietary process [30]. DNA samples were shipped back to the U.S. for analysis. All DNA samples were stored at −20°C. DNA samples from roots were diluted 1∶10 and DNA samples from soil were used undiluted for quantitative analysis with a qPCR assay as described above. All qPCR analysis plates contained negative (water) controls and quantified, positive DNA controls for consistency in relative quantification between plates. Results from qPCR analysis of similar samples (root or soil) are reported on an equivalent sample and dilution basis as cycle threshold (Ct) scores (Ct root and Ct soil), which are inversely proportional to DNA quantity in the samples.

### Data Analysis

Data were transformed before entering into the statistical analysis: top rating: arcsine (√top rating/9); root rating: arcsine (√root rate/5); and nematode numbers: log_10_(x+1). The area under the disease progress curve (AUDPC) was calculated as:
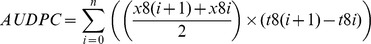
where X_i_ =  average foliar SDS severity per plot at the i^th^ observation; t_i_ =  time point of the top rating after first rating in eight-day increments at the i^th^ observation; n =  total number of observation times; in consultation with corresponding literature [41]. AUDPC provides a summary of disease severity and progression, and provides a single number for the total amount of disease a plant is suffering. Before regression analyses, data were analyzed on a per plot basis, the four factors: fumigation, watering, inoculation with *F. virguliforme*, and/or with *H. glycines* were entered into the statistical model. All analyses were conducted using the General Linear Model (PROC GLM) or procedures for mixed models (PROC MIXED) in Statistical Analysis Software System (SAS Institute, Cary, NC); data from plots with zero error variances were not included in the respective analyses. For this project, regression analyses were conducted starting with a process of analysis of the original treatment means (sixteen treatments, each replicated four times; Table S1). In a first step for data of each experimental year separately, regressions of a linear, quadratic and cubic model were analyzed for determining the relationship of one dependent and one independent variable each. In the linear regression slope comparisons between the years were done. If slopes were not significantly different the data of two years were combined. Independent parameters in pairs were entered into a multiple regression analysis, with the respective predetermined best fitting model of regression. Thus different combinations of contributions resulted. The combined contributions of two factors with their respective functions were summarized and illustrated in a three-dimensional graph. Regression analyses were done in PROC REG and PROC GLM in SAS.

## Results

### Testing of the new qPCR assay for specificity and quantification of F. virguliforme in soil

**Figure 1.Cycle:**
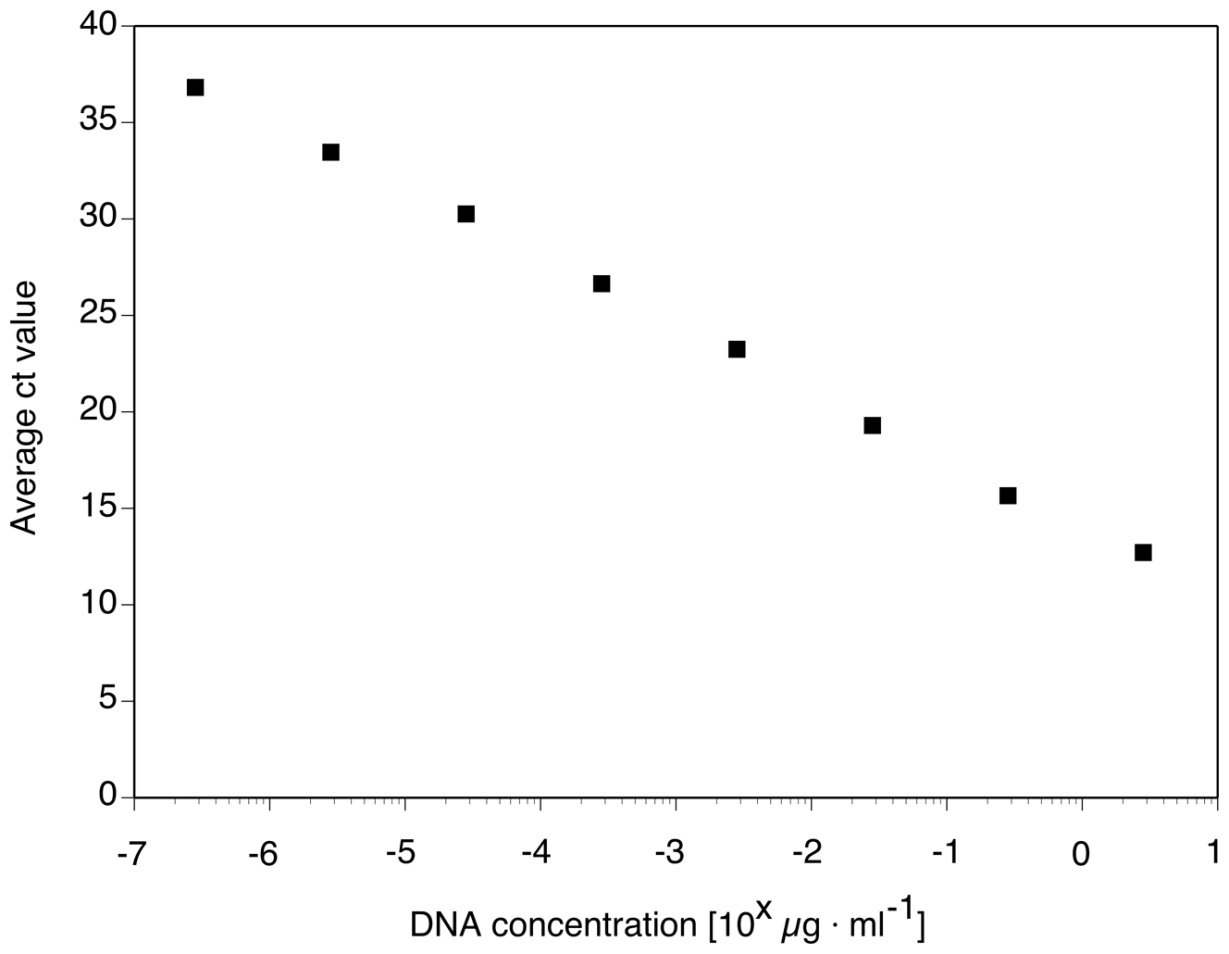
Cycle threshold (Ct)-values generated by a specific qPCR assay for a ten-fold dilution series of pure DNA of *Fusarium virguliforme*.

**Figure 2:**
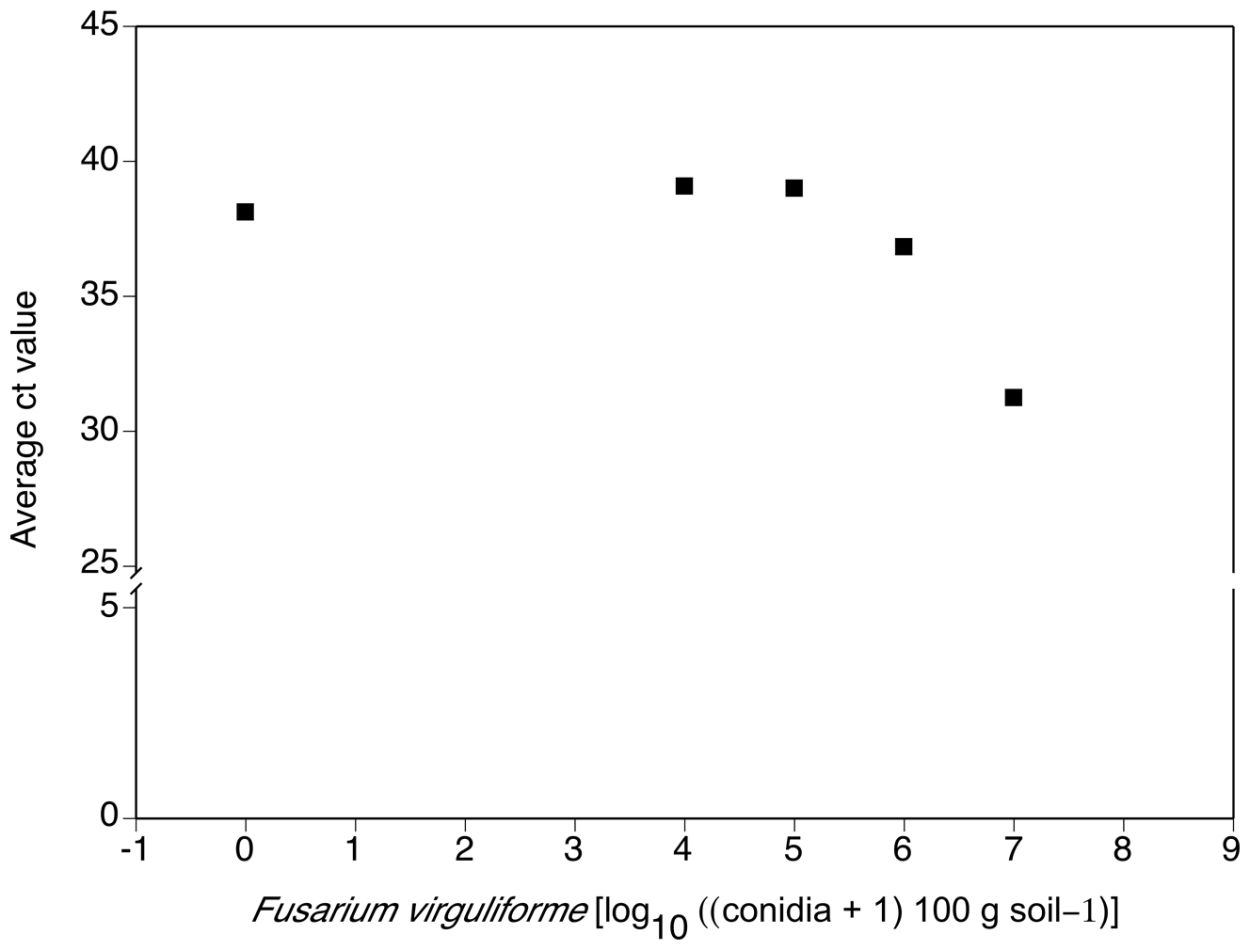
Relationship of concentrations of macroconidia of *Fusarium virguliforme* spiked into soil and quantity (inverse of Ct value) of *F. virguliforme* DNA detected with a specific qPCR assay. Samples of 500-fold dilution series of Fv macroconidia from 1×10^4^ to 1×10^7^ per 100 g of soil.

Clear quantitative relationships for pure Fv DNA dilutions vs. values measured with the qPCR assay and for Fv DNA extracted from infested soil vs. values measured, as well as specificity testing, indicated a high potential for sensitive and specific quantification of *F. virguliforme* with this new assay. For example, a close linear fit between pure Fv DNA amount and Ct values was obtained (Figure 1). The relationship of conidia added to soil and Ct value was characterized by plateau of almost no detection followed by a clear quantitative response past a threshold level (Figure 2). Tests of the qPCR assay with DNA of *Fusarium* spp. other than *F. virguliforme* and with other soilborne fungi did not result in detection (Table 1, 2). These laboratory tests of specificity for *F. virguliforme* were supported by *in silico* analysis of DNA sequences from other fungi expected in soybean and maize fields (Table 3).

**Table 3:** *In silico* (BLAST) search for primer sequences from *F. virguliforme* in the IGS sequences of other *Fusarium* spp. and other fungi expected in fields with corn and soybean production.

Fungal species	Disease; plant part	GenBank Number^a^	Blast result^b^
*Fusarium virguliforme*		AY220212^c^	48.1 (Y, 100%)
	Corn		
*Fusarium culmorum*	corn kernels	AJ854659^c^	18.3 (Y)
		AY102602^d^	24.3 (NN)
*Fusarium oxysporum*	corn kernels	JQ585778^c^	22.3 (Y)
*Fusarium proliferatum*	corn ear rots	GU737458^c^	20.3 (Y)
*Fusarium subglutinans*	ear and kernel rot	HQ165883^c^	20.3 (Y)
*Fusarium verticillioides*	ear and kernel rot	AJ880006^c^	18.3 (Y)
*Gibberella moniliforme*	grain mycoflora	HQ165881^c^	18.3 (Y)
		DQ924537^d^	24.3 (N)
*Gibberella zeae*	corn kernels	HQ165900^c^	16.4 (Y)
		XM_383254^d^	26.3 (N)
*Macrophomina phaseolina*	Nodal root fragments	N/A	N/A
*Rhizopus* sp.	Nodal root fragments	N/A	N/A
			
	Soybean		
*Calonectria crotalariae*	Red crown rot	N/A	N/A
*Diaporthe phaseolorum* var. *caulivora*	Stem canker	HM769322^c^	14.4 (N)
*Fusarium solani*	Fusarium root rot	JN041209^d^	20.3 (N)
*Macrophomina phaseolina*	Charcoal rot	GU046842^d^	24.3 (N)
*Phialophora gregata*	Brown stem rot	AF249313^c^	14.4 (N)
		AF306933^d^	18.3 (N)
*Rhizoctonia solani*	Rhizoctonia disease	GU934565^d^	22.3 (N)
*Sclerotinia sclerotiorum*	Sclerotinia stem rot	EF152636^c^	24.3 (N)
		XM_001585495^d^	32.2 (N)
*Thielaviopsis basicola*	Thielaviopsis root rot	AY997789^c^	14.4 (N)
		GQ131878^d^	18.3 (N)

a A GenBank number was provided if the search provided a reference.

b The blast search was conducted if a GenBank entry was found. The highest value of the maximum score is given.

“(Y)” The 3′ end of the forward and reverse primers at least matches 5 bases of the shown sequences separately, which means the possible success of amplification for the sequence in the database by Fv-IGS primer.

“(N)” The 3′ end of the forward and reverse primers does not match at least 5 bases of the shown sequences separately, which means very low possibility of successful amplification for the sequence in the database by Fv-IGS primer.

“(NN)” The possible amplification size is too big (>5000 bp).

c The hit sequences were in IGS region.

d The hit sequences were not in IGS region, and they were in other nuclear regions.

“N/A” not applicable because no matches were deposited in GenBank.

### Severity of foliar SDS symptoms and root necrosis and population densities of Heterodera glycines

**Figure 3:**
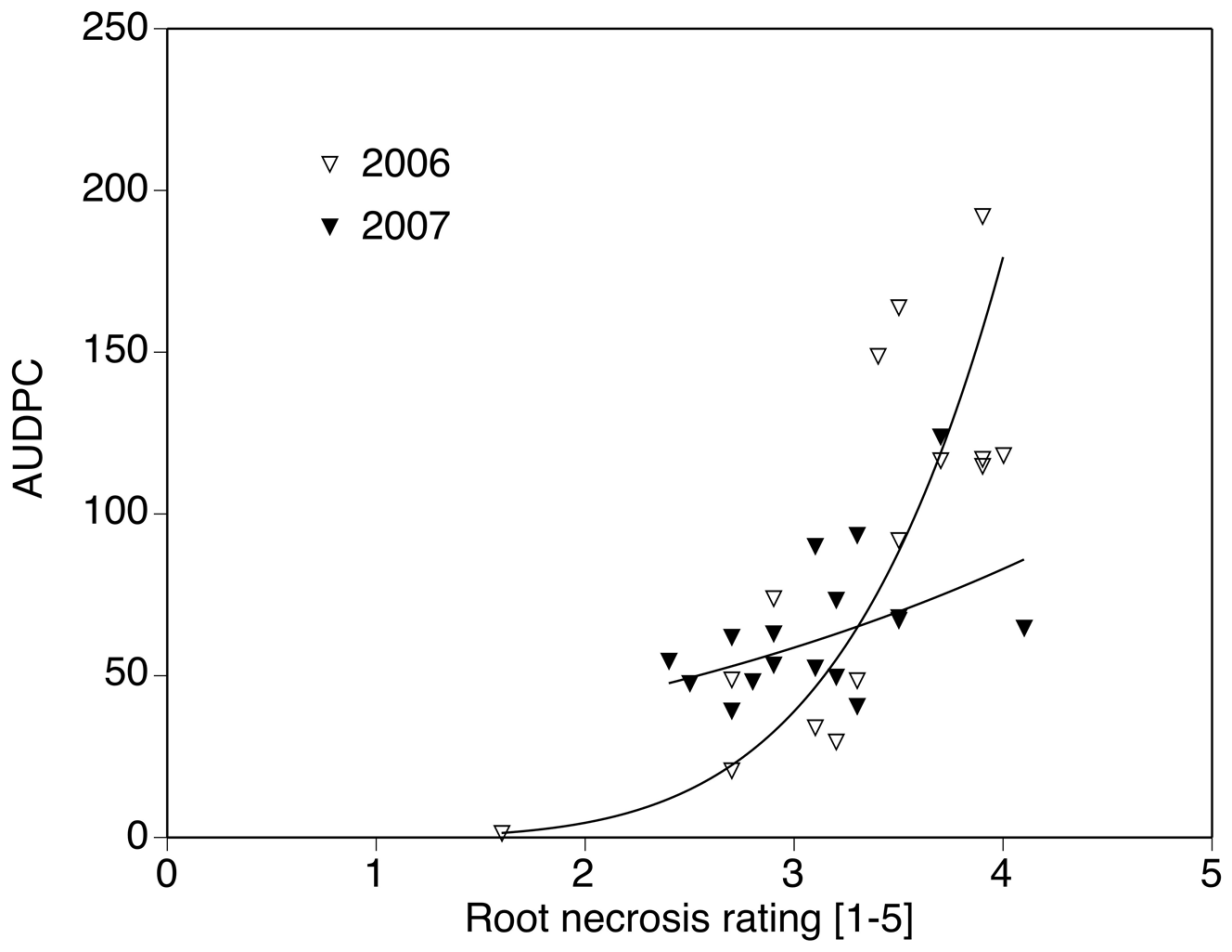
Relationship of foliar SDS severity development (AUDPC) and root necrosis rating in microplots in 2006 and 2007.

In both years, root necrosis and foliar symptom severity were positively related (Figure 3). There appeared to be less overall variability in 2007 than in 2006 for minimum to maximum values of AUDPC and root necrosis rating values. Treatment means of egg populations of *H. glycines* at planting ranged from 100 to 11,400 (log-transformed: 1.9 to 4.0) in 2006 and from 300 to 8,300 (log-transformed: 2.4 to 3.3) in 2007. At harvest, these means ranged from 600 to 2,600 (log-transformed: 2.7 to 3.4) in 2006 and from 2000 to 15,000 (log-transformed: 3.3 to 4.1) in 2007. These numbers were viewed as long-term treatment effects. In the scope of this paper, these numbers are discussed as disease inducing components of the disease complex only.

### SDS disease severity on the foliage and roots

**Figure 4:**
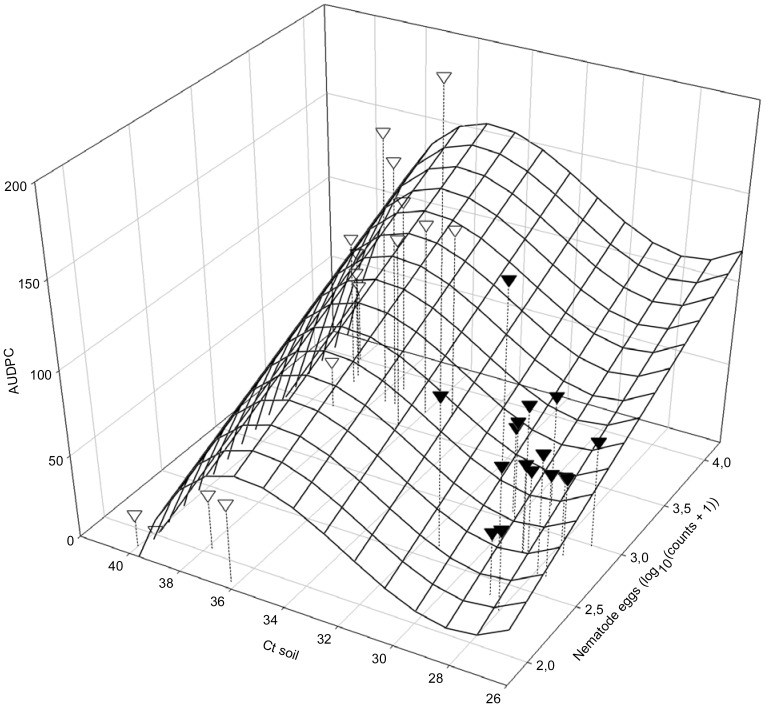
Value of AUDPC in relation to soil DNA of *Fusarium virguliforme* (Ct soil) and population densities of *Heterodera glycines* (LogPi*_H.__glycines_*) at planting: AUDPC  = 7340.30312+34.96268 LogPi*_H.__glycines_* −717.74671 Ct soil +23.01140 Ct soil^2^ −0.24258 Ct soil^3^; *R* ^2^ = 0.6804; *P*i = 0.0035; *P*a<0.01; *P*b = 0.0024; *P*c = 0.0024; ∇: 2006; ▾: 2007.

**Figure 5:**
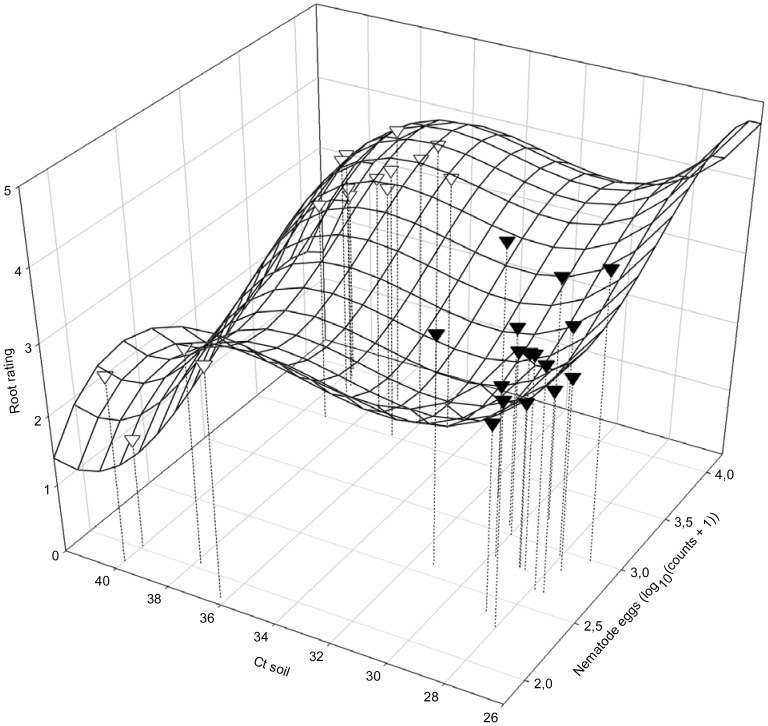
Root rating (RR) in relation to soil DNA of *Fusarium virguliforme* (Ct soil) and *Heterodera glycines* (LogPi*_H.__glycines_*) at planting: RR  = 185.20784 −22.24517 LogPi*_H.__glycines_* +7.50496 LogPi*_H.__glycines_* ^2^ −0.80092 LogPi*_H.__glycines_* s^3^ −14.74404 Ct soil +0.44699 Ct soil^2^ −0.00449 Ct soil^3^; *R^2^* = 0.6289; *P*i<0.01; *P*a = 0.0509; *P*b = 0.0504; *P*c = 0.0552; *P*d<0.01; *P*e<0.01; *P*f<0.01; ∇: 2006; ▾: 2007.

The amount of SDS disease expressed as AUDPC of the SDS foliar symptom severity was predicted by the inverse amount of DNA of *F. virguliforme* and the log-transformed population densities of *H. glycines* in soil at planting (Ct soil, LogPltEggs, respectively) by a multiple regression function (AUDPC  = 7340.30312+34.96268 LogPltEggs −717.74671 Ct soil +23.01140 Ct soil^2^ −0.24258 Ct soil^3^; *R* ^2^ = 0.6804; *P*i = 0.0035; *P*a<0.01; *P*b = 0.0024; *P*c = 0.0024; Figure 4). Similarly, the root necrosis severity rating was related to the inverse amount of DNA of *F. virguliforme* and log-transformed population densities of *H. glycines* in soil at planting (Ct soil, LogPltEggs, respectively; RR = 185.20784–22.24517 LogPltEggs +7.50496 LogPltEggs^2^ −0.80092 LogPltEggs^3^ −14.74404 Ct soil +0.44699 Ct soil^2^ −0.00449 Ct soil^3^; *R^2^* = 0.6289; *P*i<0.01; *P*a = 0.0509; *P*b = 0.0504; *P*c = 0.0552; *P*d<0.01; *P*e<0.01; *P*f<0.01; Figure 5).

### Yield in relation to disease measures

**Figure 6:**
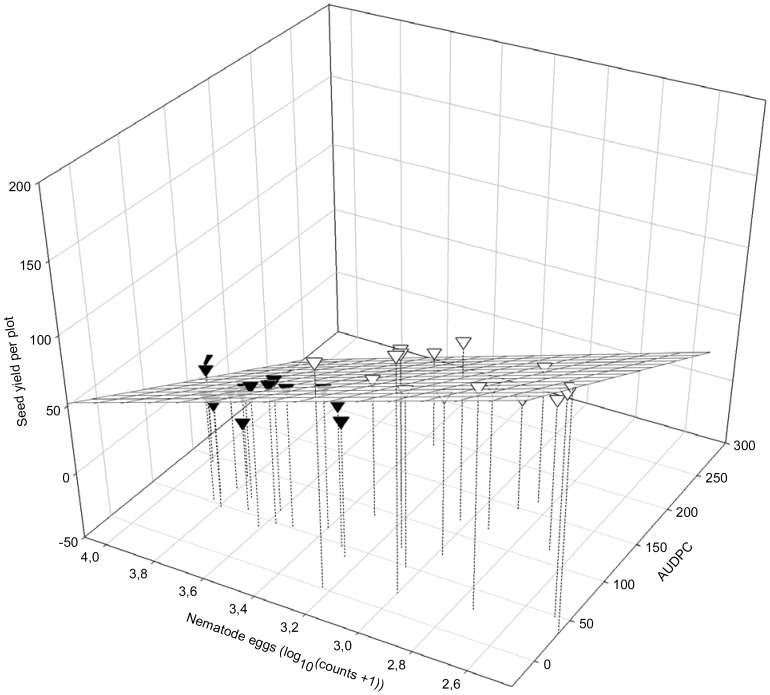
Seed yield (SY) in relation to the SDS foliar symptom severity (AUDPC) and final population densities of *Heterodera glycines* (LogPf*_H. glycines_*): SY = 280.54 - 0.366 AUDPC - 58.61 LogPf*_H. glycines_*; *R^2^* = 0.8197; *P*i = 0.0230; *P*a<0.01; *P*b<0.01; *P*c<0.01; ∇: 2006; ▾: 2007.

**Figure 7:**
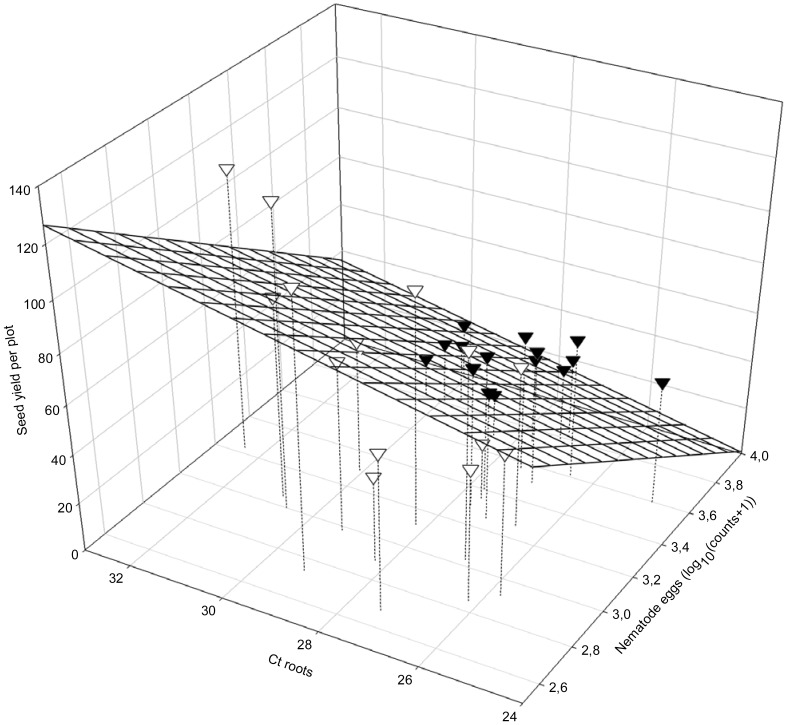
Seed yield (SY) in relation to DNA of *Fusarium virguliforme* in soybean roots (Ct roots) and final populations of *Heterodera glycines* (LogPf*_H. glycines_*): SY = 149.37667+3.88978 Ct roots - 60.42695 LogPf*_H. glycines_*; *R* ^2^ = 0.6177: *P*i = 0.0230; *P*a = 0.0819; *P*b<0.01; ∇: 2006; ▾: 2007.

**Figure 8:**
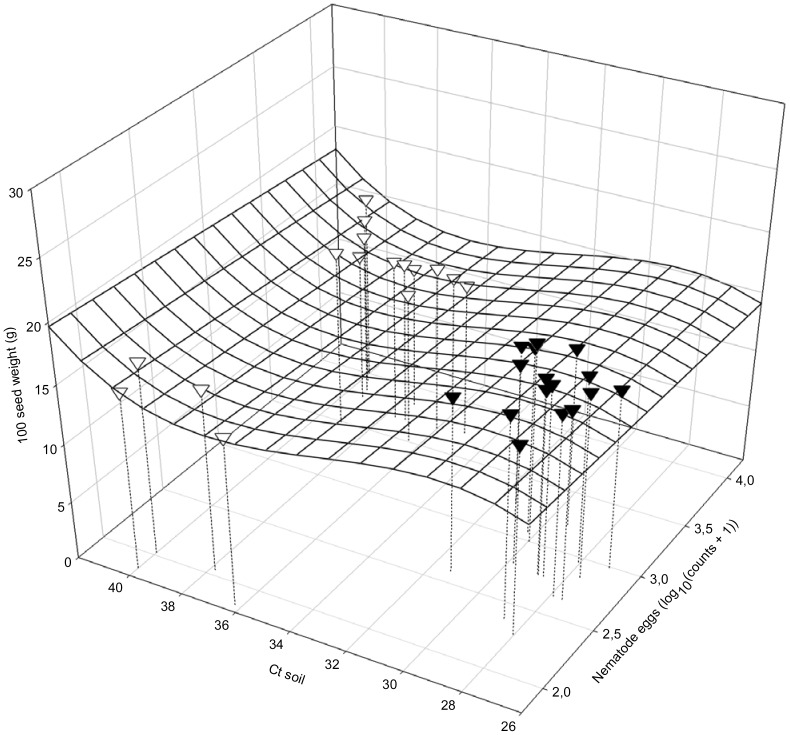
Relationship of soybean one hundred seed-weight (HSW) to initial content of soil DNA of *Fusarium virguliforme* (Ct soil) and populations of *Heterodera glycines* (LogPi*_H. glycines_*) at planting: HSW  = -323.21105 - 0.69267 LogPi*_H. glycines_* +32.08269 Ct soil - 0.99382 Ct soil^2^ +0.01012 Ct soil^3^; *R* ^2^ = 0.6630; *P*i = 0.0071; *P*a = 0.0080; *P*b = 0.0033; *P*c = 0.0024; *P*d = 0.0018; ∇: 2006; ▾: 2007.

Seed yield was dependent on the amount of foliar SDS symptoms (AUDPC) and the final egg populations of *H. glycines* (Pf*_H.__glycines_*; yield  = 280.54–0.366 AUDPC - 58.61 Pf*_H.__glycines_*; R^2^ = 0.8197; *P*<0.01; Figure 6). The seed yield also depended on the interactive relationship between DNA of *F. virguliforme* in roots (Ct roots) and final population densities of *H. glycines* at harvest (LogHarvEggs; yield  = 149.37667+3.88978 Ct roots - 60.42695 LogHarvEggs; *R* ^2^ = 0.6177: *P*i = 0.0230; *P*a = 0.0819; *P*b<0.01; Figure 7). The yield parameter of 100-seed weight (HSW) depended on initial *F. virguliforme* in soil (Ct soil) and *H. glycines* in soil at planting (LogPi*_H.__glycines_*; HSW  = −323.21105−0.69267 LogPi*_H.__glycines_* +32.08269 Ct soil - 0.99382 Ct soil^2^ +0.01012 Ct soil^3^; Figure 8), and the AUDPC and final population densities of *H. glycines* (Pf*_H. glycines_*; HSW  = 13.43 - 0.02317 AUDPC +1.05717 Pf*_H. glycines_*; *R^2^* = 0.6319; *P*<0.01, data not shown). Plant top dry weights had similar dependencies on the severity of SDS foliar symptoms (AUDPC) and final population densities of *H. glycines* (Pf*_H. glycines_*; topdry weight  = 834.14436 - 1.14584 AUDPC - 157.73526 Pf*_H. glycines_*; *R^2^* = 0.6964; *P*<0.01, data not shown).

## Discussion

### Model of SDS severity and yield effects

The current investigation provided a detailed assessment of the interactive contributions of *F. virguliforme* and *H. glycines* in the damage potential of this disease complex to soybean based on disease symptom measurements and quantification of the fungal and nematode pathogen in soil and soybean roots. The interrelationship of the pathogens in causing the disease complex was not linearly related but was more complex. Combinations of DNA of *F. virguliforme* and egg counts of the nematode in soil at planting predicted the risk for severe SDS in this study, although the environmental conditions during the growing season also can influence disease development [8]. Yield parameters were related to final amounts of disease expression. Typically initial population densities of plant-parasitic nematodes were used to model the nematodes damage potential. But in the current case, the combination of AUDPC of foliar disease and final population densities of *H. glycines* was helpful to understand their combined effects on yield. The qPCR assay was highly sensitive and specific and thus provided a novel tool for fungal population assessments and disease predictions.

### Quantitative contributions of Fusarium virguliforme and Heterodera glycines to SDS

No complex interactive parameter matrix for *H. glycines* and *F. virguliforme* was available previously. Rupe and coworkers [42] estimated depth distribution of the fungus and the nematode under the soybean crop but did not model their impacts. They found a correlation of SDS severity and population densities of the fungal pathogen, the latter determined by soil dilution plating on semi-selective media [39]. Management recommendations for plant-parasitic nematodes have relied heavily on the use of the action threshold level concept [43], [44]. No attempts to determine threshold levels of SDS under field conditions have been published. In related studies of Fusarium wilt of chickpea, caused by *Fusarium oxysporum* f. sp. *ciceris,* a steady increase of inoculum potential led to a maximum plateau at an optimum temperature and inoculum densities [45], [46]. Bhatti and Kraft [47] reported this optimum to be at inoculum densities of 500 to 1000 propagules g^-1^ of soil and around 25 to 30°C. Constraints of the current study are the limited quantitative variability of the different factors applied to the disease system. A greater diversity within these factors could perhaps be achieved under greenhouse conditions. In SDS under controlled conditions, increasing spore numbers of *F. virguliforme* used for inoculations caused increasingly more severe root necrosis [12], [48]. The need for field experiments to elucidate the interaction of a plant-parasitic nematode and a fungal disease as claimed by Evans and Haydock [cited in 49] along with difficulties of inducing consistent foliar SDS symptoms on adult plants under greenhouse conditions made it paramount to use data as generated in the current study and modeling approach.

### Biological limitations of the experimental context

These experimental plots had been infested a year prior to these data collections, and allowed for some natural fungal and nematode reproduction in that year along with some microbial community development in the soils, making infestations somewhat natural and not freshly added from artificial amendments. No record of the form *F. virguliforme* in soil was available but presumably the fungus survived as chlamydospores. The standardization of the qPCR assay was done with fungal macroconidia added to test soil. Thus the DNA amounts predicted an equivalent to a distinct number of macroconidia. In *Fusarium*, different spore types have different inoculum potential [50], [51], which added uncertainty to the current evaluations. DNA extractions were done from soybean tap roots since their infection is considered critical in overall SDS development and foliar symptom expression [52]. Strong correlations between disease and the amount of DNA in roots were found. Surprisingly, very high amounts of the fungus in soil at planting resulted in limited SDS disease development in this study. On the contrary, low quantities of the fungus were sufficient to cause severe disease if the nematode was present at high population densities. This emphasized the critical role of *H. glycines* in SDS development under the environmental and edaphic conditions of the present study. A similar observation was made in studies of early dying of potato where frequencies of the pathogens involved in a disease complex were reported; it appeared that low fungal population densities were effective in causing the disease complex in the presence of the nematode [53], [54]. This supported our hypothesis for the need of concomitant quantitative detection of both pathogens for forecasting SDS disease severity and its influence on yield.

### Specificity of the new qPCR assay based on IGS sequences of the pathogen

The illustrated qPCR assay was based on IGS sequences of the pathogen quantitatively and specifically detected only *F. virguliforme* in plants and soil. It was tested against DNA from pure cultures of multiple isolates of sixteen different *Fusarium* species, including *F. virguliforme* and other *Fusarium* species that are reported to cause SDS on soybean. The assay was also found to be specific when tested against ten other genera of fungi and Oomycetes that are common pathogens of soybean or are common in soils and plants in corn-soybean rotations in the Midwestern U.S.A. [55]. Thus, this primer set was robust for DNA evaluations under field conditions, something previously published assays did not allow to the same extent. This new qPCR assay is more specific for Fv than were similar assays that were available when this work was conducted, and provides another tool with much value for specific and sensitive detection of *F. virguliforme* in plants and soil, and for studies of the biology of this pathogen and its interactions with soybean and SCN.

### Sensitivity of the new qPCR assay based on IGS sequences of the pathogen

The modeling outcome of this project emphasized the importance of a highly sensitive and accurate qPCR assay for specific and sensitive detection of *F. virguliforme.* The qPCR assay allowed detection of DNA of *F. virguliforme*, in amounts comparable to those of similar assays for the detection of other fungal pathogens [56], [57]. The real time PCR of *F. virguliforme* from soil was quantitative over a range of 4 orders of magnitude, comparable to assays of the closely related *Fusarium solani* f. sp. *phaseoli* [58]. High sensitivity, or the ability to detect small quantities of Fv DNA, is critical for a useful qPCR assay. A standard curve to test the ability of the qPCR assay to detect serial dilutions of pure genomic DNA from *F. virguliforme* was developed. As little as 20 fg of DNA was detected when using 34 as our maximum cycle threshold (Ct). This limit of detection is comparable to that reported from many other fungal studies. A maximum Ct value of 34 was used for detection of *F. virguliforme* because a higher Ct value would decrease specificity and increase the risk for false positives. We also determined the detection limit of *F. virguliforme* macroconidia in spiked soil samples to be approximately 1,000 macroconidia per gram of soil. Thus, we achieved the goal of developing a specific, sensitive, and robust qPCR assay for detection and quantification of *F. virguliforme* that can also be used to measure fungal biomass development and improve the understanding of disease development. Similar approaches have improved the understanding of the etiology of other Fusarium diseases [57], [59]. This novel qPCR assay overcomes the challenges with detection and quantification of this soybean pathogen using semi-selective culture media.

### Conclusions

In summary, we provided a model for the assessment of disease severity and risk of SDS based on the quantification of two pathogens involved in this disease complex. This model provides a conceptual framework to elucidate this disease complex further. We also report a new specific and sensitive qPCR assay for *F. virguliforme* designed for further study of this challenging plant disease complex. A better understanding of the interplay of the causal agent and its facilitator will improve disease management and breeding efforts in combating this disease complex.

## Supporting Information

Table S1 **Average treatment responses to the factors (i) non-treated or fumigated, (ii) non-infested or infested with** ***Fusarium virguliforme*** **, (iii) non-infested or infested with** ***Heterodera glycines*** **, and (iv) natural precipitation or additional weekly watering in amount of foliar SDS disease (AUDPC), root rating (RR), log_10_–transformed population densities of** ***Heterodera glycines*** **at planting (LogPi) and harvest (LogPf) of soybean ’Williams 82’ in microplots in 2006 and 2007; inverse quantities of DNA of** ***Fusarium virguliforme*** **in soil at planting (Ct soil) and roots at full seed growth stage R6 (Ct roots); seed yield (SY) and weight of 100 seeds (HSW) along with plant topdry weight (DW).**
(DOC)
