# Supplementary material for: Contributions of Fusarium virguliforme and Heterodera glycines to the Disease Complex of Sudden Death Syndrome of Soybean
Source: PLoS One. 2014 Jun 16;9(6):e99529. doi: 10.1371/journal.pone.0099529 (PMC4059700; doi:10.1371/journal.pone.0099529)
Supplement: Table S1 — Average treatment responses to the factors (i) non-treated or fumigated, (ii) non-infested or infested with Fusarium virguliforme , (iii) non-infested or infested with Heterodera glycines , and (iv) natural precipitation or additional weekly watering in amount of foliar SDS disease (AUDPC), root rating (RR), log10–transformed population densities of Heterodera glycines at planting (LogPi) and harvest (LogPf) of soybean ’Williams 82’ in microplots in 2006 and 2007; inverse quantities of DNA of Fusarium virguliforme in soil at planting (Ct soil) and roots at full seed growth stage R6 (Ct roots); seed yield (SY) and weight of 100 seeds (HSW) along with plant topdry weight (DW). (DOC) [file pone.0099529.s001.doc]

**Table S1.** Average treatment responses to the factors (i) non-treated or fumigated, (ii) non-infested or infested with *Fusarium virguliforme*, (iii) non-infested or infested with *Heterodera glycines*, and (iv) natural precipitation or additional weekly watering in amount of foliar SDS disease (AUDPC), root rating (RR), log_10_–transformed population densities of *Heterodera glycines* at planting (LogPi) and harvest (LogPf) of soybean ’Williams 82’ in microplots in 2006 and 2007; inverse quantities of DNA of *Fusarium virguliforme* in soil at planting (Ct soil) and roots at full seed growth stage R6 (Ct roots); seed yield (SY) and weight of 100 seeds (HSW) along with plant topdry weight (DW).

|  |  | | | |  | |  | |  | |  | |  |
| --- | --- | --- | --- | --- | --- | --- | --- | --- | --- | --- | --- | --- | --- |
|  | Parameters | | | | Disease | | *Heterodera glycines* | | *Fusarium virguliforme* | | Yield | | Top |
| YEAR | (i) | (ii) | (iii) | (iv) | AUDPC | RR | LogPi | LogPf | Ct soil | Ct roots | SY | HSW | DW |
|  |  |  |  |  |  |  |  |  |  |  |  |  |  |
| 2006 | - | - | - | - | 48.4 | 2.7 | 4.00 | 3.05 | 40.00 | 30.85 | 82.3 | 16.0 | 231.7 |
| 2006 | - | + | - | - | 163.4 | 3.5 | 3.68 | 2.85 | 37.65 | 26.20 | 54.1 | 14.2 | 162.3 |
| 2006 | - | - | + | - | 73.5 | 2.9 | 3.93 | 2.98 | 39.78 | 29.29 | 69.8 | 14.8 | 227.8 |
| 2006 | - | + | + | - | 148.4 | 3.4 | 3.68 | 2.89 | 37.25 | 28.26 | 35.5 | 14.2 | 182.3 |
| 2006 | - | - | - | + | 66.7 | 3.5 | 3.84 | 3.12 | 39.45 | 28.21 | 95.0 | 14.1 | 281.4 |
| 2006 | - | + | - | + | 116.5 | 3.9 | 3.69 | 2.92 | 36.03 | 25.78 | 58.4 | 14.5 | 202.6 |
| 2006 | - | - | + | + | 29.2 | 3.2 | 3.49 | 2.74 | 39.10 | 29.20 | 111.4 | 15.4 | 314.5 |
| 2006 | - | + | + | + | 114.4 | 3.9 | 3.51 | 2.68 | 36.45 | 27.39 | 64.0 | 13.2 | 258.7 |
| 2006 | + | - | - | - | 0.9 | 1.6 | 2.02 | 3.23 | 40.00 | 32.43 | 114.1 | 16.5 | 356.9 |
| 2006 | + | + | - | - | 33.6 | 3.1 | 2.04 | 2.44 | 37.83 | 27.35 | 126.2 | 15.6 | 428.1 |
| 2006 | + | - | + | - | 91.4 | 3.5 | 3.77 | 3.30 | 39.35 | 30.08 | 54.7 | 13.0 | 164.3 |
| 2006 | + | + | + | - | 191.6 | 3.9 | 3.87 | 3.35 | 36.08 | 27.59 | 23.7 | 12.3 | 99.2 |
| 2006 | + | - | - | + | 20.3 | 2.7 | 1.87 | 3.00 | 40.00 | 30.58 | 122.0 | 15.3 | 476.4 |
| 2006 | + | + | - | + | 48.1 | 3.3 | 1.85 | 2.49 | 36.23 | 27.08 | 112.9 | 14.4 | 426.0 |
| 2006 | + | - | + | + | 117.7 | 4.0 | 3.84 | 3.27 | 37.50 | 26.60 | 65.7 | 12.5 | 277.5 |
| 2006 | + | + | + | + | 116.1 | 3.7 | 3.72 | 3.03 | 35.03 | 26.86 | 84.7 | 13.4 | 325.1 |
| 2007 | - | - | - | - | 61.5 | 2.7 | 3.00 | 3.84 | 29.97 | 29.67 | 24.6 | 17.2 | 138.3 |
| 2007 | - | + | - | - | 89.6 | 3.1 | 2.77 | 3.82 | 28.84 | 30.41 | 15.4 | 16.0 | 101.8 |
| 2007 | - | - | + | - | 47.2 | 2.5 | 2.80 | 3.79 | 29.83 | 27.28 | 30.3 | 17.1 | 139.0 |
| 2007 | - | + | + | - | 54.1 | 2.4 | 2.78 | 3.70 | 28.90 | 28.47 | 18.0 | 16.8 | 110.9 |
| 2007 | - | - | - | + | 52.0 | 3.1 | 2.77 | 3.51 | 28.66 | 27.02 | 55.6 | 16.5 | 241.7 |
| 2007 | - | + | - | + | 47.8 | 2.8 | 2.85 | 3.30 | 27.64 | 27.65 | 57.7 | 16.0 | 202.3 |
| 2007 | - | - | + | + | 38.7 | 2.7 | 2.38 | 3.26 | 28.66 | 27.19 | 52.8 | 17.4 | 233.3 |
| 2007 | - | + | + | + | 62.6 | 2.9 | 2.65 | 3.40 | 27.58 | 27.69 | 56.3 | 16.2 | 210.7 |
| 2007 | + | - | - | - | 123.4 | 3.7 | 3.27 | 4.09 | 31.41 | 30.25 | 10.8 | 13.9 | 86.9 |
| 2007 | + | + | - | - | 73.0 | 3.2 | 2.65 | 3.81 | 27.89 | 29.95 | 25.3 | 15.7 | 86.8 |
| 2007 | + | - | + | - | 52.9 | 2.9 | 3.07 | 3.84 | 30.27 | 27.24 | 31.8 | 16.1 | 201.0 |
| 2007 | + | + | + | - | 67.6 | 3.5 | 3.22 | 3.73 | 29.34 | 27.71 | 35.7 | 15.5 | 141.3 |
| 2007 | + | - | - | + | 93.0 | 3.3 | 2.59 | 3.98 | 31.33 | 30.08 | 23.2 | 15.1 | 167.9 |
| 2007 | + | + | - | + | 49.2 | 3.2 | 2.29 | 3.57 | 28.03 | 27.45 | 56.0 | 16.1 | 255.2 |
| 2007 | + | - | + | + | 40.1 | 3.3 | 2.96 | 3.61 | 28.06 | 26.51 | 57.4 | 16.1 | 239.5 |
| 2007 | + | + | + | + | 64.3 | 4.1 | 2.99 | 3.60 | 27.07 | 24.76 | 50.7 | 15.4 | 224.5 |

1. Non-treated (-) or preinfestation-fumigated with methylbromide in 2005 (+)
2. Non-infested (-) or infested with *Fusarium virguliforme* in 2005 (+)
3. Non-infested (-) or infested with *Heterodera glycines* in 2005 (+)
4. Natural precipitatation (-) or weekly watering in 2005, 2006, and 2007 (+)
